# Insight into the Relationship Between Motor and Cognitive Symptoms in Essential Tremor

**DOI:** 10.1007/s12311-024-01704-y

**Published:** 2024-05-15

**Authors:** Giulia Paparella, Luca Angelini, Roberta Margiotta, Massimiliano Passaretti, Daniele Birreci, Davide Costa, Antonio Cannavacciuolo, Martina De Riggi, Danilo Alunni Fegatelli, Matteo Bologna

**Affiliations:** 1 Department of Human Neurosciences, Sapienza University of Rome, Rome, Italy; 2 IRCCS Neuromed, Pozzilli, IS, Italy; 3 Department of Public Health and Infectious Disease, Sapienza University of Rome, Rome, Italy

**Keywords:** Essential tremor, Essential tremor-plus, Bradykinesia, Cognitive impairment, Neuropsychology

## Abstract

Essential tremor (ET) is a heterogeneous disorder characterized by bilateral upper limbs action tremor and, possibly, neurological signs of uncertain significance, including voluntary movement abnormalities and cognitive disturbances, i.e., the so-called ‘soft’ signs configuring the ET-plus definition. While motor and cognitive disturbances often coexist in ET, their interrelationship remains largely unexplored. Here we aim to further investigate the relationship between motor symptoms, objectively assessed through kinematic analysis, and cognitive dysfunctions in ET. Seventy ET patients underwent clinical examination, as well as kinematic recordings of tremor and finger tapping and a thorough cognitive assessment. We then tested clinic-demographic and kinematic differences between patients with and without cognitive abnormalities, i.e., with mild cognitive impairment (MCI). Correlation analysis served to explore potential associations between kinematic and cognitive data. Forty-three ET patients (61.42%) had MCI. ET-MCI patients exhibited reduced movement velocity during finger tapping compared to those with normal cognition (*p* < 0.001). Lower movement velocity during finger tapping was associated with poorer cognitive performance. Namely, we observed a correlation between movement velocity and performance on the Babcock Story Immediate and Delayed Recall Test (*r* = 0.52 and *r* = 0.45, both *p* < 0.001), as well as the interference memory task at 10 and 30 s (*r* = 0.3, *p* = 0.008 and *r* = 0.2, *p* = 0.03). In this study, we have provided data for a better pathophysiological interpretation of motor and cognitive signs in ET, including the role played by the cerebellum or extra-cerebellar areas, which possibly underpin both signs.

**Supplementary Information:**

The online version contains supplementary material available at 10.1007/s12311-024-01704-y.

## Introduction

Essential tremor (ET) is a heterogeneous condition defined by the presence of bilateral action tremor of the upper limbs [1], and possibly, various motor and non-motor symptoms, i.e., ‘soft signs,’ configuring the ET-plus definition [1]. In this context, individuals with ET may exhibit a mild slowing of voluntary movement, often referred to as bradykinesia [2–13], and the impaired movement performance cannot be solely attributed to the secondary effects of tremor [3–7, 9–11]. Furthermore, various studies have documented the presence of cognitive dysfunction in ET patients [2, 7, 8, 13–21]. For instance, recent findings indicate that over 30% [8], and possibly up to 50% of ET patients [7] exhibit mild cognitive impairment (MCI) based on neurophysiological assessments [22, 23]. The predominant subtypes of MCI in ET are amnestic multi-domain MCI (aMCI-md) and non-amnestic single-domain MCI (naMCI-sd) [7, 8], with memory and executive function being the most affected cognitive domains.

Various potential pathophysiological mechanisms have been suggested to explain motor and cognitive disturbances in ET, including dysfunction in both the cerebellum and extra-cerebellar areas. Data from animal and human studies demonstrate that some movement parameters, including velocity, are encoded in cerebellar neural activity [24, 25], and that cerebellar diseases, including ET, are associated with slowed movement execution [3, 12, 26]. Nevertheless, additional mechanisms may be implicated. These include an altered oscillating activity in the wider cerebral network involving the thalamus and the primary motor cortex (M1) [3, 26, 27], as well as subtle changes in basal ganglia dopaminergic activity [3, 6]. Given the established role of the cerebellum in ET, cerebellar dysfunction may also play a role in the pathophysiology of cognitive abnormalities in this condition [15, 17, 28]. As the cerebellar output involves significant projections to cerebral association areas, notably the prefrontal cortex, damage to the cerebellum may give rise to noticeable impairments in cognitive capabilities [15, 29–31]. Neurodegeneration of additional brain regions, however, such as the thalamus and frontal areas, may also contribute to cognitive changes in ET [8, 15, 32, 33].

To our knowledge, no previous study has specifically investigated the relationship between motor and cognitive dysfunction in ET with the help of kinematic techniques for objective movement analysis. Investigating this issue in ET, with the understanding that motor and cognitive dynsfuction often occur in combination in patients [1, 2, 7, 19, 34], could provide data for a proper pathophysiological interpretation of these abnormalities, including the role played by the cerebellum, which possibly underpins both signs [3, 8, 28, 35]. In the present study, we aim to explore the relationship between tremor and movement performance, as objectively assessed with kinematic analysis, and cognitive functions, evaluated with a comprehensive neuropsychological assessment in a broad sample of ET patients. We aimed to get further insight into a common pathophysiological denominator between motor and cognitive aspects in ET.

## Materials and Methods

### Participants

Seventy right-handed patients (29 females − 41.4%) diagnosed with ET and ET-plus [1] were consecutively recruited from the movement disorder outpatient clinic of the Department of Human Neurosciences, Sapienza, University of Rome. Patients had an isolated tremor syndrome of bilateral upper limb action tremor, with at least 3 years’ duration, with or without tremor in other locations, with or without additional neurological signs of uncertain significance (soft signs), including rest tremor, questionable bradykinesia, questionable dystonic posturing, impaired tandem gait, and/or mild memory impairment [1]. Exclusion criteria included the evidence of other prominent neurological signs suffice to make alternative diagnoses, such as dystonia, ataxia, or parkinsonism [1, 36, 37]. None of the members of the initial sample met the criteria for a diagnosis of dementia [38]. The mean age ± standard deviation (SD) was 68.27 ± 13.54 years (range: 20–86 years) (Table 1). Patients underwent one experimental session, including a thorough neurological examination with the Fahn-Tolosa-Marin Tremor Rating Scale (FTM-TRS) and the Movement Disorder Society-sponsored revision of the Unified Parkinson’s Disease Rating Scale (MDS-UPDRS) Part III, as well as kinematic recordings and neuropsychological assessment. Patients undergoing tremor treatment had their evaluations conducted after a 48-hour medication discontinuation [at the time of the experimental evaluations, 35 patients were on therapy and 35 were not on any therapy. Among the treated patients, 25 were taking Propranolol (ranging daily dose: 10–120 mg), 6 were under Clonazepam (0.4–1.8 mg/daily), 2 patients were taking Primidone (500–750 mg/daily), one patient was taking a combination of Clonazepam (1.8 mg/daily) and Propranolol (80 mg/daily), and one was under Topiramate (75 mg/daily) and Propranolol (100 mg/daily)]. The experimental procedures adhered to the principles of the Declaration of Helsinki and received approval from the local ethics committee. Written informed consent was obtained from all participants prior to their involvement in the study.

**Table 1:** Clinical, demographic and kinematic data in patients with essential tremor (ET). Patients were classified in two groups according to the neuropsychological evaluation: ET patients with normal cognition (ET-NC), and ET patients with mild cognitive impairment (ET-MCI). Age, age at tremor onset, and tremor duration are expressed in years (y). Fahn-Tolosa-Marin Tremor Rating Scale (FTM-TRS), Movement Disorder Society-sponsored revision of the Unified Parkinson’s Disease Rating Scale (MDS-UPDRS). UL: upper limbs, CI: curvature index, CV: coefficient of variation, N. mov: number of movements. Data are indicated as median (interquartile range). P values from Fisher’s exact test and Mann-Whitney U test. Significant values are shown in bold

	ET-NC (27)	ET-MCI (43)	*P* values
**Clinical data**			
Sex	10 F/17 M	19 F/24 M	0.557
Age (y)	72 (13)	72 (15.5)	0.171
Age of tremor onset (y)	52 (29)	52 (19)	0.141
Tremor duration (y)	13 (13)	10 (12.5)	0.453
Family history	15Y/12 N	25Y/18 N	0.659
FTM-TRS	20 (21)	20 (17)	0.391
MDS-UPDRS III	6 (7)	7.5 (6)	0.487
**Kinematic data**			
*Tremor*			
Postural UL (GRMS^2)	0.08 (0.12)	0.08 (0.07)	0.50
Postural UL (Hz)	5.61 (1.53)	5.89 (1.56)	0.7
Kinetic UL (CI)	1.05 (0.05)	1.04 (0.03)	0.21
Rest UL (GRMS^2)	0.03 (0.02)	0.02 (0.02)	0.51
Rest UL (Hz)	5.91 (1.47)	6.04 (1.35)	0.43
Head (GRMS^2)	0.12 (0.06)	0.1 (0.07)	0.64
Head (Hz)	4.43 (2.41)	4.43 (2)	0.18
*Finger tapping*			
N. Mov	43 (15.91)	39.5 (19.16)	0.3
CV	0.1 (0.04)	0.09 (0.04)	0.32
Amplitude (degrees)	41.78 (18.52)	45.51 (15.47)	0.61
Velocity (degrees/sec)	1066.12 (280.75)	809.4 (384.85)	**< 0.001**
Amplitude decrement (degree/n mov)	-0.04 (0.12)	-0.09 (0.2)	0.4
Velocity decrement (degree/sec)/n mov	-2.66 (2.83)	-3.05 (4.12)	0.59

### Kinematic Recordings and Analysis

The assessment was conducted as in previous works [4–8, 39–42]. We used a 3-D optoelectronic system (SMART motion system, BTS, Milan, Italy) with three infrared cameras detecting movement in three-dimensional space, and the analysis was carried out offline using dedicated software (SMART Analyzer, BTS Engineering, Italy). Reflective markers were strategically placed on the dominant hand, and on the distal phalanx of the index finger and thumb. We recorded postural tremor in two different arm positions: arms outstretched in front of the chest (Posture 1 - P1) and arms flexed at the elbows in a lateral “wing beating” posture (Posture 2 - P2) [4–8, 39, 41, 42]. Three 45-second recordings were obtained for each position. Additionally, three 15-second recordings were taken for each arm to assess kinetic tremor during arm movements (‘pointing task’) [4–8, 39, 41, 42]. Again, three 45-second recordings were conducted to assess rest tremor while patients were seated comfortably on a chair with their arms fully relaxed [4–8, 39, 41, 42]. Head tremor was also recorded. Tremor was quantified by measuring the root mean square (RMS) of the acceleration traces, expressed in GRMS^2. Fast Fourier Transform was also applied to the accelerometry traces to calculate the power spectrum, and the tremor frequency peak (expressed in Hz) [4–8, 39, 41, 42]. Patients’ mean values from the right and left sides were considered for the analyses. We utilized a specialized algorithm for the analysis of kinetic tremor during arm movement. Specifically, we computed the curvature index (CI) of the arm endpoint trajectory [4–8, 39, 41, 42]. The CI indicates movement homogeneity, with higher values indicating the greater kinetic tremor [4–8, 39, 41, 42]. Regarding postural tremor data, we averaged data from P1 and P2. Again, the patients’ mean values from the right and left sides were considered for the analyses. Also, we recorded finger-tapping, a commonly employed task in clinical practice for assessing bradykinesia in patients. Participants were instructed to perform 15 s of repetitive finger movements as quickly and extensively as possible. Three 15-s recordings were performed with a 60-s pause between recordings [4–7, 39, 40]. The finger-tapping movement velocity (degrees per second) was determined using linear regression techniques [4–7, 39, 40]. We also measured the movement rhythm, specifically the coefficient of variation (CV), calculated as the standard deviation divided by the mean value of the inter-tap intervals [4–7, 39, 40], and other movement parameters, as detailed elsewhere [4–7, 39, 40].

### Neuropsychological Assessment

Neuropsychological assessment was conducted by experienced neuropsychologists, who were blinded to the kinematic data [7, 8]. Global cognition was assessed by the Montreal Cognitive Assessment (MoCA) [43, 44]. The assessment of specific cognitive domains included the evaluation of executive function (Coloured Progressive Matrices, the Stroop Colour and Word test, phonemic verbal fluency task, semantic verbal fluency task, Frontal Assessment Battery – FAB, Trail Making Test B), attention (Trail Making Test A, Visual Search), memory (Babcock Story Recall Test (BSRT), The Rey Auditory Verbal Learning Test (RAVLT), memory with interference task, Forward Digit Span, Backward Digit Span), and visuo-constructional abilities (Rey-Osterrieth Complex Figure test). Raw scores were corrected for age and years of education using normative data available for each test. Based on the standardization cut-offs of each test, calculated at ≤ 2 standard deviations (SD) from the average for correct responses and ≥ 2 SD for time responses and errors, the performances were considered normal or impaired. Two impaired executive function tests were required for a patient’s executive function domain to be considered impaired, while only one impaired test was required for all the other domains. ET patients with no impairment or impairment in only one test were classified as having normal cognition (ET-NC). ET patients with MCI were then classified as amnestic MCI (aMCI) and non-amnestic (naMCI). Finally, patients with an impairment in at least 50% of the tests in one domain or impairment in at least 50% of the tests in ≥ 2 domains were considered to have single-domain (MCI-sd) or multi-domain MCI (MCI-md), respectively [7, 8, 22, 23].

### Statistical Analysis

Categorical variables were presented as frequencies and compared using Fisher’s exact test. Mann-Whitney U tests were employed for assessing quantitative clinical and kinematic data in ET-NC and ET-MCI. Additionally, we conducted a subgroup analysis, comparing data among ET-NC and the subtypes of ET-MCI (aMCI and naMCI) using the Kruskal-Wallis analysis of variance (ANOVA). Post-hoc comparisons were performed using the Dunn’s test. Spearman’s correlation analysis was conducted to explore potential relationships between clinical and kinematic data. All results are presented as median (interquartile range) unless otherwise specified. The level of significance was set at *p* < 0.05. Results were corrected for multiple comparisons using the false discovery rate (FDR) [45]. Data analysis was carried out using STATISTICA® (TIBCO Software Inc., Palo Alto, California, USA) and implemented in R (R version 4.2.2. R Foundation for Statistical Computing, Vienna, Austria. URL https://www.R-project.org/).

## Results

### Clinical Data and Neuropsychological Results on the Whole Patients Sample

All patients, by definition, had bilateral action tremor of the upper limbs. We found rest tremor in 13 out of 70 patients (19%)v, questionable bradykinesia in 12/70 patients (17%), and rest tremor plus questionable bradykinesia in 10 out of 70 patients (14%) although no patient fully met the criteria for parkinsonism [36]. Eleven patients (15.7%) exhibited questionable dystonic posturing. Finally, 10 out of 70 participants (14.3%) exhibited a slightly impaired tandem gait. In total, 26 patients (37.1%) displayed at least one motor soft sign.

**Fig. 1:**
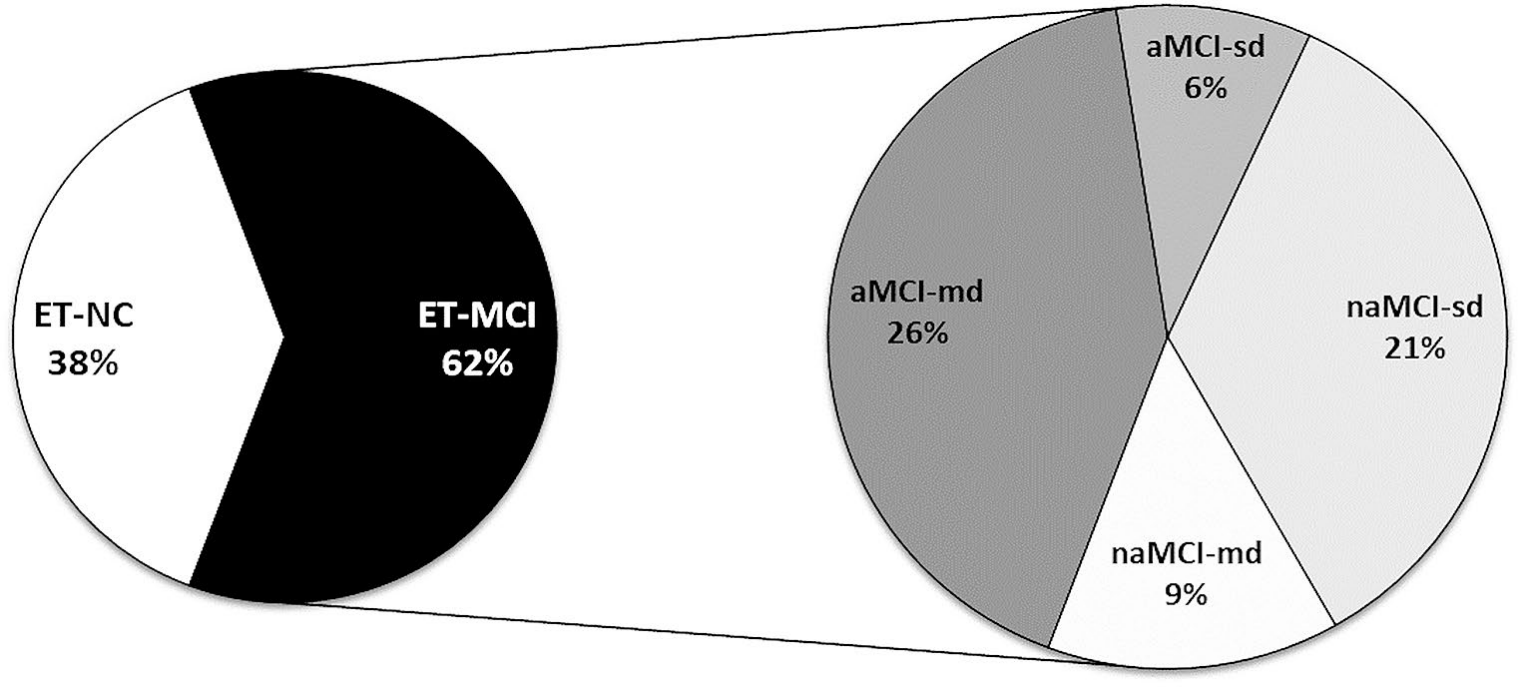
Neuropsychological results in patients with essential tremor (ET). ET patients with no impairment or impairment in only one test were classified as having normal cognition (ET-NC). ET patients with mild cognitive impairment (MCI) were classified as amnestic MCI (aMCI) and non-amnestic (naMCI). Patients with an impairment in at least 50% of the tests in one domain or impairment in at least 50% of the tests in ≥ 2 domains were considered to have single-domain (MCI-sd) or multi-domain MCI (MCI-md), respectively

Neuropsychological evaluation revealed that 27 patients (38.17%) fell into the ET-NC category, while 43 (61.42%) were categorized as ET-MCI (refer to Table 1; Fig. 1). We observed no differences in age, age at tremor onset, and tremor duration between ET-NC and ET-MCI patients (all p values > 0.05). Similarly, the prevalence of a positive family history and FTM-TRS and MDS-UPDRS Part III scores was comparable between the two groups (Table 1). Within the ET-MCI group, 22 patients had aMCI (4 with aMCI-sd and 18 with aMCI-md), and 21 patients had naMCI, with 15 having naMCI-sd and 6 having naMCI-md (see Fig. 1). Detailed neuropsychological results are provided in Supplementary Table 1. No significant differences in clinical data were identified between ET-NC, aMCI, and naMCI and patients except for age, which was significantly higher in the naMCI subgroup (Supplementary Table 2).

In summary, 57 out of 70 patients (81.4%) were classified as ET-plus due to motor and/or non-motor soft signs. Supplementary Table 3 depicts detailed cognitive scores in ET-plus patients and in patients without any soft additional signs.

### Kinematic Data Analysis: ET-NC vs. ET-MCI

The amplitude and frequency of postural and rest tremor in the upper limbs and the head, along with the severity of kinetic upper limb tremor, expressed by the CI, did not differ between ET-NC and ET-MCI patients (all p values > 0.05) (Table 1). Kinematic analysis also revealed that ET-MCI patients were slower than ET-NC when performing repetitive finger tapping (*p* < 0.001) while showing no other movement abnormalities (Table 1).

As additional analyses, we examined finger-tapping movement velocity values in ET patients with questionable dystonia compared to those without questionable dystonia. We found no significant differences between these two subgroups [median finger tapping velocity in patients with questionable dystonia (interquartile range): 1009.89 (261.63) degrees/s; without questionable dystonia: 889.72 (374.41) degrees/s, p: 0.48]. Finally, we investigated possible differences in finger-tapping performance between ET patients with an impaired tandem gait, compared to those with a normal tandem gait. Again, we found no differences between the two subgroups [1009.9 (262.35) vs. 922.95 (379.13) degrees/s, p: 0.67).

### Kinematic Data Analysis on ET-MCI Subtypes

**Fig. 2:**
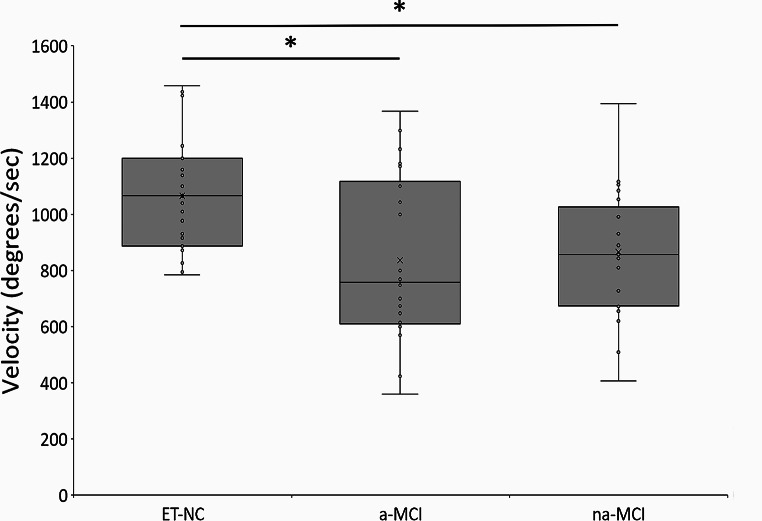
Kinematic results of finger-tapping movements in patients with essential tremor (ET). NC: normal cognition. aMCI: amnestic mild cognitive impairment. naMCI: non-amnestic MCI. Finger-tapping movement velocity are expressed in degrees/sec. Kinematic values were compared using Kruskal-Wallis analysis of variance (ANOVA). Asterixes indicate significant values at post-hoc comparisons

The amplitude and frequency of postural and rest tremor in the upper limbs and the head, along with the severity of kinetic upper limb tremor, showed no significant differences between the subgroups (all p values > 0.05) (Supplementary Table 2). The analysis of finger-tapping velocity revealed significant differences in terms of movement velocity among the subgroups [H_2_ = 11.86, *p* = 0.003] (Supplementary Table 2). Post-hoc comparisons indicated that patients with aMCI and those with naMCI exhibited slower movement velocity than ET-NC patients (p values = 0.005 and 0.02, respectively), with no other significant differences observed between aMCI and naMCI (*p* = 1) (Fig. 2).

### Correlation Analysis Between Kinematic Data and Cognitive Scores

**Fig. 3:**
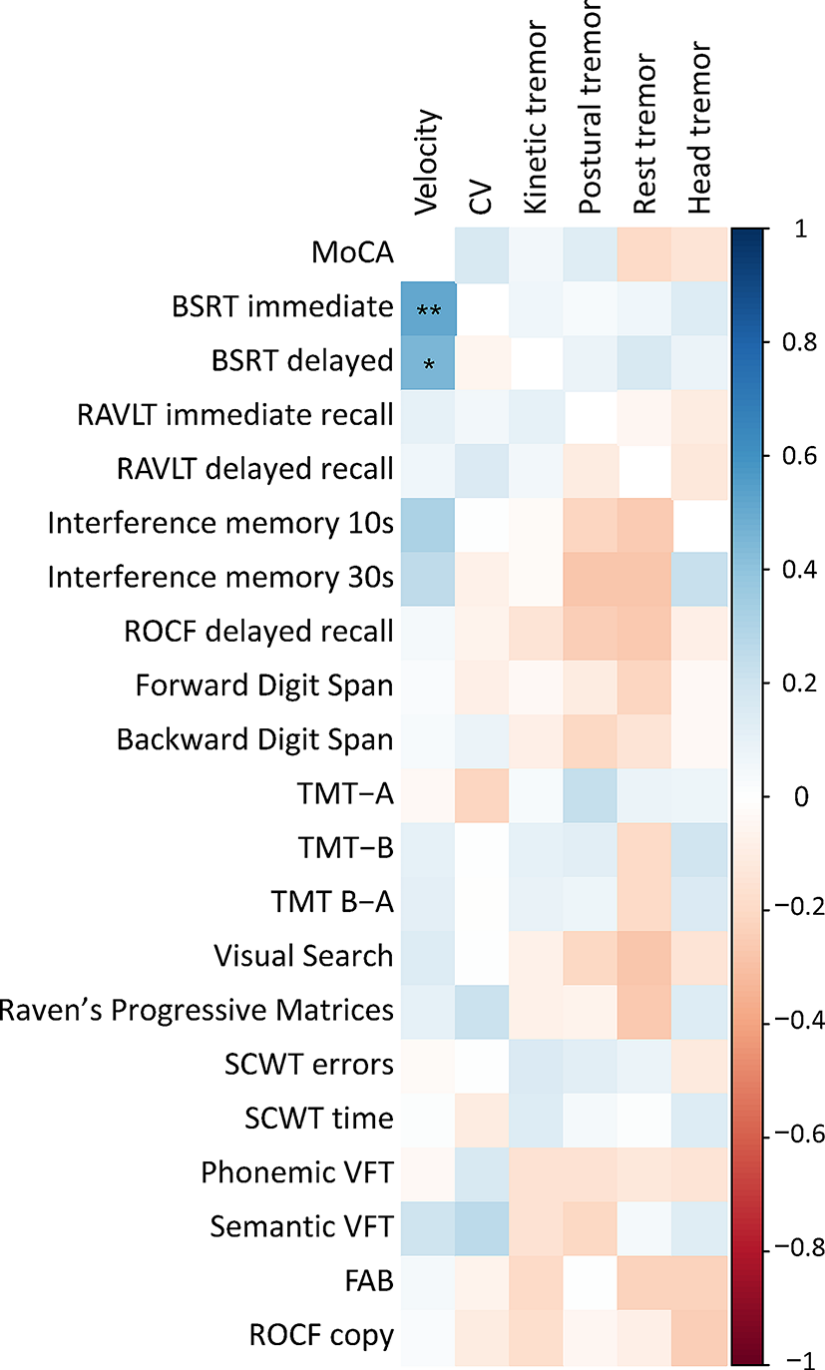
Correlation matrix between cognitive and kinematic variables. The colour map indicates matrices of Spearman’s correlations and coefficients, asterixis for significant p-values adjusted for false discovery rate (FDR) (**:*p* < 0.01, *:*p* < 0.05). Please note that a positive correlation was also observed between finger-tapping velocity and interference memory at 10 and 30 s, revealing correlation coefficients of 0.3 (*p* = 0.008) and 0.2 (*p* = 0.03), respectively, although these were not significant after FDR correction. CV: coefficient of variation. MoCA: Montreal Cognitive Assessment, BSRT: Babcock Story Recall Test, RAVLT: Rey-Auditory Verbal Learning Test, ROCF: Rey–Osterrieth complex figure, TMT-A: Trail Making Test-A, TMT-B: Trail Making Test-B, TMT-BA: Trail Making Test B – A, SCWT: Stroop Color and Word Test, VFT: Verbal Fluency Test, FAB: Frontal Assessment battery

We found a positive correlation between movement velocity during finger tapping and the Babcock Story Immediate and Delayed Recall Test (BSRT) scores, showing a correlation coefficient of 0.52 (*p* < 0.001, p-adj = 0.001) and 0.45 (*p* < 0.001, p-adj = 0.01), respectively. Additionally, we observed a positive correlation with interference memory at 10 and 30 s, revealing correlation coefficients of 0.3 (*p* = 0.008) and 0.2 (*p* = 0.03), respectively, although these results were not significant after FDR correction (Fig. 3, Supplementary Table 4, Supplementary Fig. 1). These findings suggest that lower movement velocity corresponds to poorer performance on tests demanding both memory and executive skills. No other significant correlations were observed between clinical, kinematic, and other cognitive data (all p values > 0.05) (Fig. 3, Supplementary Table 4), including between FTM-TRS total scores and cognitive parameters (r ranging from − 0.28 to 0.32, p-adj ranging from 0.16 to 0.96).

## Discussion

In this study, we employed clinical and kinematic analyses to objectively assess tremor and repetitive finger movements, a widely employed test for bradykinesia assessment in clinical practice, in a comprehensive sample of ET patients who underwent thorough neuropsychological evaluations. Our findings support that isolated movement slowness is a common soft sign in ET [2–7, 9–11, 40]. Our findings also confirmed that a substantial proportion of ET had MCI [2, 7, 8, 14–21]. Again, the aMCI-md was the most common form of ET-MCI [7, 8, 22, 23]. As recently highlighted [14], due to the ET clinical heterogeneity, the relatively small number of patients and the different cognitive tests used in the various studies, it was not possible to draw firm conclusions on the specific cognitive involvement in ET. Attention, executive functions, verbal memory, language, and global cognitive function seemed to be consistently affected in previous reports [14]. Our data further demonstrate that memory and executive functions are the most involved cognitive domains in ET. Importantly, we observed that ET-MCI exhibited slowed finger-tapping compared to patients with normal cognition. Furthermore, a correlation was identified between movement velocity during finger tapping and cognitive performance in memory and executive functions—indicating that lower movement velocity is associated with more pronounced deficits in these specific cognitive domains. Conversely, we did not observe any correlations between tremor and cognitive functions, nor between movement velocity during finger tapping and tremor severity. These results offer insight into the pathophysiology of motor and non-motor manifestations in ET, and into the concept of ET-plus.

The most innovative aspect of this study is the comprehensive investigation of motor and non-motor symptoms relationship in ET, which is still unclear [2, 5, 7, 8, 13, 18, 34]. Our findings show that individuals with ET-MCI exhibit slower motor performance than those with preserved cognitive functions. Namely, the velocity of finger-tapping correlated with performance on tests evaluating verbal memory (BSRT) and memory with interference, i.e., the ability to hierarchically organize and store information and maintain memory information after an interfering working memory task. These tests tap into memory abilities, working memory, and executive functions. It is established that episodic memory involves the recruitment of the medial temporal lobe, particularly the hippocampus, and the prefrontal cortex (PFC) [46, 47], which is crucial in organizing information for efficient storage [48]. The medial temporal lobe and the left inferior PFC also support interference resolution in verbal memory [49]. Moreover, cerebellar activation has been demonstrated during tests assessing attention and response selection or inhibition [50], which are both important for the memory with interference test. Consistently, other studies have shown cerebellar involvement in dual tasks as well as a substantial cerebellar contribution in executive functions [29, 51]. Overall, the observed correlation between slowed movement velocity during finger tapping and deficits in BSRT and memory with interference yields valuable insights into ET pathophysiology. These findings could be interpreted through two major hypotheses.

One hypothesis posits that the correlation between voluntary movements and cognitive alterations in ET stems from a shared pathophysiological mechanism. Specifically, it is proposed that the cerebellum, a key player in the pathophysiology of ET [8, 15, 28, 33, 35, 52], may underlie both the slowed movement execution and cognitive decline observed in patients. This hypothesis gains support from the established role of the cerebellum in controlling specific movement parameters, including movement velocity [3, 24–26]. Additionally, various studies have highlighted associations between degenerative cerebellar disease, cerebellar tumours, ischemic lesions, and movement slowness in ET [3]. Further supporting the role of the cerebellum in altered voluntary movement execution, we have recently demonstrated that patients with tremor induced by valproate may also present movement slowness which reflect a cerebellar network disruption due to valproate [39]. Moreover, cerebellar dysfunction may give rise to various cognitive dysfunctions, including the Cognitive Affective Cerebellar Syndrome (CCAS), as postulated by Schmahmann et al. in the ‘90s. The CCAS is thought to result from impaired cerebellar connections with cortical associative areas, namely the frontal and parietal cortex, language areas, and the limbic lobe [15, 17, 29, 31]. The breakdown of these connections could lead to various cognitive impairments in ET. For example, frontal connections abnormalities could lead to dysexecutive functions, parietal connection impairment might cause visuospatial dysfunctions, associative area involvement might result in language impairment, and connections with the limbic lobe could explain psychiatric abnormalities in ET patients [14]. Recent evidence emphasizes the role of the dentate nucleus in these connections, with reduced connectivity linked to both tremor severity and cognitive impairment [52]. However, it is important to note that our study did not reveal correlations between tremor, as clinically and kinematically assessed, and cognitive decline, nor between tremor and slowed movement execution. Such correlations would have supported the cerebellar hypothesis underlying both motor and non-motor disorders in ET [18]. In this regard, we also failed to find a correlation between the severity of head tremor and the cognitive decline, in contrast to previous studies that had shown in patients with ET and head tremor a higher frequency of non-motor symptoms indicating a more severe cerebellar dysfunction [8, 34]. Furthermore, no significant differences in finger-tapping movement velocity were observed between ET patients with impaired tandem gait (suggesting again greater cerebellar involvement) and those without such abnormality [18, 53]. Overall, the data seem to argue against the hypothesis that posits a correlation between voluntary movements and cognitive alterations in ET solely relying on cerebellar involvement. Accordingly, the cerebellar pathology may be unable to explain the full burden and progression of cognitive impairment seen in ET [15].

An alternative hypothesis that warrants consideration is the ‘extracerebellar hypothesis.’ Hence, the relationship between motor and cognitive abnormalities in ET may be rooted in extracerebellar, more diffuse alterations encompassing various brain areas and circuits. Such a hypothesis aligns with both (i) the notion that bradykinesia likely arises from network dysfunction rather than the dysfunction of a singular brain area [3, 12, 26], and (ii) the established understanding that memory is a complex cognitive function with diffuse physiological bases in the brain. Hence, it is plausible that a more intricate involvement of brain structures beyond the cerebello-thalamo-cortical pathway contributes to cognitive function in ET [15, 21, 32, 54]. This notion is consistent with prior evidence demonstrating impaired circuitry in frontal regions in ET patients with attentive dysfunction [55]. Neuroimaging data corroborate this hypothesis, revealing that specific cognitive domains affected in ET patients, such as poorer performance on verbal fluency tests, are associated with bilateral frontal hypoperfusion [56]. Increased connectivity in resting state networks has also been linked to worse performance on diverse cognitive domains in ET [57]. Moreover, studies have highlighted that ET patients with MCI exhibit widespread white and grey matter alterations involving the insular lobe, medial frontal gyrus, cingulate regions, and frontal and parietal lobes, along with alterations in the cerebellum-frontal pathway [32, 54]. Involvement of the hippocampus and parahippocampal regions has also been demonstrated in ET [32, 58], together with alterations in volumes of subcortical nuclei, which are not limited to the motor domain and include structures involved in cognitive and behavioral functions. [59]. Finally, some pathological studies demonstrated in ET a neurodegeneration affecting other brain areas besides the cerebellum [15, 32, 33].

In line with the ‘extra cerebellar hypothesis’, one could speculate that motor and cognitive dysfunctions in ET are due to the parallel involvement of brain areas crucial for controlling both cognitive and motor functions. Finally, the ‘extracerebellar hypothesis’ would support the concept that ET, as in the case of other neurological diseases [3, 26], should be considered a network disorder, in which the cerebellum plays a relevant, but not unique role.

It is important to acknowledge the possible study confounding and limitations. Regarding the risk of misdiagnosis, the patients in our study underwent extensive long-term follow-up in the outpatient clinic, strengthening our confidence in the accuracy of the clinical diagnosis of ET. Significantly, none of the patients met the criteria for parkinsonism. Indeed, among the 70 patients evaluated clinically, only 10 exhibited rest upper limbs tremor, accompanied by slight movement slowness. However, the presence of bradykinesia in these individuals was questionable, and there was no history of progressively worsening parkinsonism based on the clinical assessment. Again, in instances of diagnostic uncertainty (8 patients out of 10), we conducted a DAT scan using single-photon emission computed tomography (SPECT), and all findings indicated no significant dopaminergic dysfunction, thereby substantiating the absence of alternative conditions. For patients undergoing tremor treatment, evaluations were carried out after a 48-hour medication discontinuation, guaranteeing the exclusion of any substantial therapeutic influence on our results. However, possible effects of long-term intake of medications such as primidone and topiramate have to be considered when interpreting our results. Larger-scale studies are required to strengthen and validate our findings because the sample size could potentially influence the results, especially in analysing smaller subgroups. Lastly, it is well known that subjects with MCI may present slight bradykinesia and other mild parkinsonian signs regardless of the presence of tremor [60, 61]. In the present study we did not include data from non-ET patients with and without MCI, as it would go beyond the scope of our work. Further studies are needed to clarify this issue.

## Conclusion

Our kinematic investigation of voluntary movements in ET patients, coupled with a thorough neuropsychological assessment, has unveiled a significant connection between motor and cognitive impairment in ET. These findings contribute to a deeper understanding of the pathophysiological mechanisms in ET. Importantly, our results carry potential implications for clinical and therapeutic approaches. If future studies continue to substantiate the pathophysiological basis of the observed relationship, it could ameliorate targeted intervention strategies addressing both motor and non-motor aspects in affected individuals.

## Electronic Supplementary Material

Below is the link to the electronic supplementary material.

Supplementary Material 1

## Data Availability

The data supporting this study’s findings are available on request from the corresponding author.

## Abbreviations

ANOVA: Analysis of Variance
BSRT: Babcock Story Recall Test
CCAS: Cognitive Affective Cerebellar Syndrome
CV: Coefficient of Variation
CI: Curvature Index
ET: Essential Tremor
ET-NC: Essential Tremor with Normal Cognition
FAB: Frontal Assessment Battery
FDR: False Discovery Rate
MCI: Mild Cognitive Impairment
aMCI: Amnestic Single-Domain MCI
aMCI-md: Amnestic multi-domain MCI
naMCI-sd: Non-Amnestic Single-Domain MCI
naMCI-md: Non-Amnestic Multi-Domain MCI
M1: Primary Motor Cortex
FTM-TRS: Fahn-Tolosa-Marin Tremor Rating Scale
MDS-UPDRS: Movement Disorder Society-Sponsored Revision of the Unified Parkinson’s Disease Rating Scale
MoCA: Montreal Cognitive Assessment
P1: Posture 1
P2: Posture 2
SD: Standard Deviation
