# Supplementary material for: Insight into the Relationship Between Motor and Cognitive Symptoms in Essential Tremor
Source: Cerebellum. 2024 May 15;23(5):2050–9. doi: 10.1007/s12311-024-01704-y (PMC11489240; doi:10.1007/s12311-024-01704-y)
Supplement: Supplementary file 1 — Supplementary Material 1 [file 12311_2024_1704_MOESM1_ESM.docx]

| **Cognitive domains - neuropsychological test** | **aMCI-sd (4)** | **a-MCI-md (18)** | **naMCI-sd (15)** | **naMCI-md (6)** | **ET-NC (27)** |
| --- | --- | --- | --- | --- | --- |
| **Global Cognition** |  |  |  |  |  |
| MoCA [≤15,5] | 24.9 (2.5) | 24.0 (2.5) | 24.6 (2.8) | 22.3 (3.9) | 25.6 (4.0) |
| **Verbal Memory** |  |  |  |  |  |
| BSRT immediate [≤3,09] | 5.6 (1.9) | 2.9 (3.0) | 5.6 (1.1) | 3.4 (0.4) | 5.5 (1.9) |
| BSRT delayed [≤ 2,38] | 6.8(2.5) | 2.7(2.7) | 6.7(1.9) | 4.2(1.2) | 6.3(2.3) |
| RAVLT immediate recall [≤28,52] | 46.4(13.9) | 31.8(5.9) | 43.0(12.6) | 34.0(8.1) | 43.2(9.1) |
| RAVLT delayed recall [≤4,68] | 11.1 (2.4) | 5.3 (3.6) | 8.9 (3.6) | 5.8 (0.6) | 9.9 (2.9) |
| Interference memory 10s | 8.0 (2.8) | 8.5 (2.8) | 8.0(3.0) | 7.0 (0.0) | 9.0 (1.0) |
| Interference memory 30s | 8.5 (1.5) | 5.5 (4.5) | 7.0 (3.0) | 5.0 (3.0) | 8.0 (2.0) |
| **Visuospatial memory** |  |  |  |  |  |
| ROCF delayed recall [≤6,33] | 7.2 (3.6) | 11.0 (5.3) | 18.8 (4.9) | 14.9 (7.7) | 21.0 (11.0) |
| **Short-term/ working memory** |  |  |  |  |  |
| Forward Digit Span [≤4,25] | 5.9 (0.5) | 5.4 (0.9) | 5.7 (0.7) | 4.9 (0.3) | 5.4 (1.2) |
| Backward Digit Span [≤2,64] | 5.4 (0.9) | 4.1(1.5) | 4.4 (1.1) | 3.9 (1.2) | 4.7 (1.4) |
| **Attention** |  |  |  |  |  |
| TMT-A [>93] | 39.9 (4.2) | 35.0 (16.3) | 37.2 (21.3) | 65.1 (52.3) | 33.4 (11.5) |
| Visual Search [≤30] | 44.1 (2.1) | 44.6 (8.4) | 55.5 (4.0) | 38.5 (10.8) | 52.4 (8.0) |
| **Executive functions** |  |  |  |  |  |
| Raven’s Progressive Matrices [≤17,5] | 31.3 (1.3) | 28.0 (6.1) | 32.5 (4.3) | 27.0 (3.0) | 32.0 (4.3) |
| SCWT errors [>4,24] | 0.0 (0.5) | 0.4 (1.8) | 0.0 (0.7) | 0.6 (2.0) | 0.0 (0.4) |
| SCWT time [>36,92] | 20.9 (3.1) | 21.5 (11.3) | 13.8 (13.0) | 22.5 (8.3) | 18.0 (12.3) |
| Phonemic VFT [<17,35] | 31.7 (7.5) | 24.5 (8.3) | 33.9 (10.2) | 32.0 (9.0) | 34.0 (13.4) |
| Semantic VFT [≤16] | 35.0 (8.5) | 15.6 (18.2) | 40.3 (14.9) | 26.0 (10.2) | 38.0 (12.1) |
| FAB [≤13,4] | 17.3 (1.6) | 16.1 (3.0) | 17.5 (1.7) | 14.0 (5.6) | 17.2 (2.2) |
| TMT-B [>282] | 90.1 (40.7) | 36.7 (180.8) | 43.0 (84.7) | 40.0 (169.5) | 64.0 (65.8) |
| TMT B-A [>186] | 49.3 (41.9) | 32.5 (111.0) | 23.0 (60.6) | 11.0 (98.8) | 25.0 (61.0) |
| **Visuo-constructional abilities** |  |  |  |  |  |
| ROCF copy [≤23,76] | 31.3 (3.0) | 35.5 (3.3) | 34.6 (5.0) | 32.2 (7.3) | 33.0 (6.3) |

**Supplementary Table 1. Detailed neuropsychological test scores in patients with essential tremor (ET).** aMCI-sd: amnestic single-domain mild cognitive impairment, aMCI-md: amnestic multi-domain MCI, naMCI-sd: non-amnestic single-domain MCI, naMCI-md: non-amnestic multi-domain MCI, ET-NC: patients with ET and normal cognition. MoCA: Montreal Cognitive Assessment, BSRT: Babcock Story Recall Test, RAVLT: Rey-Auditory Verbal Learning Test, ROCF**:** Rey–Osterrieth complex figure, TMT-A: Trail Making Test-A, TMT-B: Trail Making Test-B, TMT-BA: Trail Making Test B – A, SCWT: Stroop Color and Word Test, VFT: Verbal Fluency Test, FAB: Frontal Assessment battery. Cut-off values are indicated in square branches. Data are indicated as median (interquartile range). All neuropsychological scores were adjusted for age and education.

|  | **ET-NC (27)** | **ET-aMCI (22)** | **ET-naMCI (21)** | **P values** |
| --- | --- | --- | --- | --- |
| **Clinical data** |  |  |  |  |
| Sex | 10F/17 M | 7F/15 M | 12F/9M | 0.08 |
| Age (y) | 72 (13) | 67.5 (9.75) | 78 (12) | **0.02** |
| Age of tremor onset (y) | 52 (29) | 57.5 (22.25) | 61 (20) | 0.08 |
| Tremor duration (y) | 13 (13) | 10 (20.5) | 10 (10) | 0.75 |
| Family history | 15Y/12N | 14Y/8N | 11Y/10N | 0.33 |
| FTM-TRS | 20 (21) | 18 (14.47) | 21 (16) | 0.57 |
| MDS-UPDRS III | 6 (7) | 7 (6) | 6 (10) | 0.28 |
| **Kinematic data** |  |  |  |  |
| *Tremor* |  |  |  |  |
| Postural UL (GRMS^2) | 0.08 (0.12) | 0.09 (0.05) | 0.06 (0.08) | 0.24 |
| Postural UL (Hz) | 5.61 (1.53) | 5.77 (2.35) | 5.93 (0.36) | 0.74 |
| Kinetic UL (CI) | 1.05 (0.05) | 1.04 (0.03) | 1.05 (0.03) | 0.4 |
| Rest UL (GRMS^2) | 0.03 (0.02) | 0.02 (0.01) | 0.02 (0.08) | 0.44 |
| Rest UL (Hz) | 5.91 (1.47) | 6.76 (2.72) | 5.91 (0.37) | 0.65 |
| Head (GRMS^2) | 0.12 (0.06) | 0.11 (0.08) | 0.09 (0.05) | 0.07 |
| Head (Hz) | 4.43 (2.41) | 4.35 (2) | 4.33 (1.89) | 0.67 |
| *Finger tapping* |  |  |  |  |
| N Mov | 43 (15.91) | 39 (18.72) | 41 (19) | 0.59 |
| CV | 0.1 (0.04) | 0.09 (0.06) | 0.09 (0.03) | 0.33 |
| Amplitude (degrees) | 41.78 (18.52) | 48.9 (11.49) | 42.3 (19.78) | 0.34 |
| Velocity (degrees/sec) | 1066.12 (280.75) | 758.35 (462.99) | 857.55 (323.65) | **0.003** |
| Amplitude decrement (degree/n mov) | -0.04 (0.12) | -0.09 (0.16) | -0.09 (0.19) | 0.67 |
| Velocity decrement (degree/sec)/n mov (degree/n mov) | -2.66 (2.83) | -3.37 (3.8) | -3.02 (4.95) | 0.7 |

**Supplementary Table 2. Clinical, demographic and kinematic data in patients with essential tremor (ET) and normal cognition (NC), amnestic mild cognitive impairment (ET-aMCI) and non-amnestic MCI (ET-naMCI).** Age, age at tremor onset and tremor duration are expressed in years (y). Fahn-Tolosa-Marin Tremor Rating Scale (FTM-TRS). Movement Disorder Society-sponsored revision of the Unified Parkinson's Disease Rating Scale (MDS-UPDRS). UL: upper limbs, CI: curvature index, CV: coefficient of variation, N. mov: number of movements. Values were compared to using Fisher's exact test and Kruskal-Wallis’s analysis of variance, when appropriate. Data are indicated as median (interquartile range). Significant values are in bold.

| **Cognitive domains - neuropsychological test** | **ET-plus (57)** | **Pure ET (13)** | **P values** |
| --- | --- | --- | --- |
| **Global Cognition** |  |  |  |
| MoCA [≤15,5] | 24.52 (3.53) | 25.59 (3.56) | 0.32 |
| **Verbal Memory** |  |  |  |
| BSRT immediate [≤3,09] | 24.52 (3.54) | 25.59 (3.56) | 0.37 |
| BSRT delayed [≤ 2,38] |  |  | 0.42 |
| RAVLT immediate recall [≤28,52] | 4.85 (3.25) | 5.50 (1.90) | **0.03** |
| RAVLT delayed recall [≤4,68] | 5.50 (3.90) | 6.30 (2.30) | 0.15 |
| Interference memory 10s | 35.30 (9.80) | 43.15 (9.05) | 0.13 |
| Interference memory 30s | 6.60 (3.29) | 9.90 (2.85) | 0.1 |
| **Visuospatial memory** | 7.50 (3.00) | 9.00 (1.00) |  |
| ROCF delayed recall [≤6,33] | 6.00 (3.00) | 8.00 (2.00) | **0.02** |
| **Short-term/ working memory** |  |  |  |
| Forward Digit Span [≤4,25] | 14.00 (8.35) | 21.00 (10.98) | 0.17 |
| Backward Digit Span [≤2,64] |  |  | 0.13 |
| **Attention** | 5.42 (1.00) | 5.37 (1.17) |  |
| TMT-A [>93] | 4.19 (1.33) | 4.73 (1.43) | 0.39 |
| Visual Search [≤30] |  |  | 0.54 |
| **Executive functions** | 38.72 (19.60) | 33.42 (11.50) |  |
| Raven’s Progressive Matrices [≤17,5] | 46.00 (14.00) | 52.38 (8.00) | 0.69 |
| SCWT errors [>4,24] |  |  | 0.41 |
| SCWT time [>36,92] | 29.75 (5.50) | 32.00 (4.25) | 0.93 |
| Phonemic VFT [<17,35] | 0.24 (1.36) | 0.00 (0.45) | 0.88 |
| Semantic VFT [≤16] | 20.50 (11.27) | 18.00 (12.33) | 0.92 |
| FAB [≤13,4] | 30.00 (10.23) | 34.00 (13.38) | 0.25 |
| TMT-B [>282] | 29.00 (23.70) | 38.00 (12.10) | 0.08 |
| TMT B-A [>186] | 16.60 (2.70) | 17.20 (2.20) | 0.06 |
| **Visuo-constructional abilities** | 63.68 (108.31) | 64.00 (65.79) |  |
| ROCF copy [≤23,76] | 30.50 (76.38) | 25.00 (61.00) | 0.35 |

**Supplementary Table 3. Detailed neuropsychological test scores in patients with essential tremor and additional soft signs (ET-plus) and in patients with ET without any additional soft sign (pure ET).** MoCA: Montreal Cognitive Assessment, BSRT: Babcock Story Recall Test, RAVLT: Rey-Auditory Verbal Learning Test, ROCF**:** Rey–Osterrieth complex figure, TMT-A: Trail Making Test-A, TMT-B: Trail Making Test-B, TMT-BA: Trail Making Test B – A, SCWT: Stroop Color and Word Test, VFT: Verbal Fluency Test, FAB: Frontal Assessment battery. Cut-off values are indicated in square branches. Data are indicated as median (interquartile range) and compared with the Mann-Whitney U test. All neuropsychological scores were adjusted for age and education. Significant values are in bold.

|  |  | **Velocity** | **CV** | **Kinetic tremor** | **Postural tremor** | **Rest tremor** | **Head tremor** |
| --- | --- | --- | --- | --- | --- | --- | --- |
| **MoCA** | r | -0.069 | 0.164 | 0.055 | 0.138 | -0.2 | -0.145 |
|  | p_adj | 0.823 | 0.635 | 0.871 | 0.643 | 0.635 | 0.635 |
| **BSRT immediate** | r | 0.519 | 0.012 | 0.069 | 0.04 | 0.064 | 0.145 |
|  | p_adj | **0.001** | 0.981 | 0.823 | 0.891 | 0.889 | 0.635 |
| **BSRT delayed** | r | 0.454 | -0.051 | 0.116 | 0.083 | 0.165 | 0.088 |
|  | p_adj | **0.01** | 0.884 | 0.727 | 0.823 | 0.727 | 0.814 |
| **RAVLT immediate recall** | r | 0.108 | 0.058 | 0.102 | -0.14 | -0.05 | -0.108 |
|  | p_adj | 0.727 | 0.846 | 0.732 | 0.635 | 0.891 | 0.727 |
| **RAVLT delayed recall** | r | 0.065 | 0.158 | 0.052 | -0.107 | -0.085 | -0.127 |
|  | p_adj | 0.823 | 0.635 | 0.877 | 0.727 | 0.823 | 0.688 |
| **Interference memory 10s** | r | 0.313 | 0.005 | -0.022 | -0.214 | -0.255 | 0.213 |
|  | p_adj | 0.367 | 0.986 | 0.933 | 0.542 | 0.542 | 0.542 |
| **Interference memory 30s** | r | 0.256 | -0.078 | -0.022 | -0.273 | -0.275 | 0.229 |
|  | p_adj | 0.542 | 0.823 | 0.933 | 0.542 | 0.542 | 0.542 |
| **ROCF delayed recall** | r | 0.046 | -0.069 | -0.149 | -0.25 | -0.265 | -0.09 |
|  | p_adj | 0.89 | 0.823 | 0.635 | 0.542 | 0.542 | 0.814 |
| **Forward Digit Span** | r | 0.027 | -0.089 | -0.036 | -0.102 | -0.219 | -0.031 |
|  | p_adj | 0.915 | 0.808 | 0.891 | 0.738 | 0.635 | 0.9 |
| **Backward Digit Span** | r | 0.034 | 0.086 | -0.08 | -0.209 | -0.143 | -0.036 |
|  | p_adj | 0.897 | 0.814 | 0.823 | 0.542 | 0.727 | 0.891 |
| **TMT-A** | r | -0.037 | -0.21 | 0.037 | 0.238 | 0.088 | 0.071 |
|  | p_adj | 0.891 | 0.542 | 0.891 | 0.542 | 0.823 | 0.823 |
| **TMT-B** | r | 0.103 | 0.005 | 0.109 | 0.122 | -0.193 | 0.196 |
|  | p_adj | 0.732 | 0.986 | 0.727 | 0.727 | 0.635 | 0.542 |
| **TMT B-A** | r | 0.111 | 0 | 0.095 | 0.078 | -0.194 | 0.155 |
|  | p_adj | 0.727 | 1 | 0.767 | 0.823 | 0.635 | 0.635 |
| **Visual Search** | r | 0.149 | 0.007 | -0.071 | -0.21 | -0.278 | -0.142 |
|  | p_adj | 0.635 | 0.985 | 0.823 | 0.542 | 0.542 | 0.635 |
| **Raven’s Progressive Matrices** | r | 0.107 | 0.22 | -0.073 | -0.066 | -0.261 | 0.141 |
|  | p_adj | 0.727 | 0.542 | 0.823 | 0.823 | 0.542 | 0.635 |
| **SCWT errors** | r | -0.02 | 0.009 | 0.159 | 0.129 | 0.082 | -0.111 |
|  | p_adj | 0.934 | 0.985 | 0.635 | 0.683 | 0.828 | 0.727 |
| **SCWT time** | r | 0.013 | -0.11 | 0.15 | 0.05 | 0.011 | 0.141 |
|  | p_adj | 0.978 | 0.727 | 0.635 | 0.884 | 0.985 | 0.635 |
| **Phonemic VFT** | r | -0.037 | 0.161 | -0.159 | -0.158 | -0.126 | -0.141 |
|  | p_adj | 0.891 | 0.635 | 0.635 | 0.635 | 0.767 | 0.635 |
| **Semantic VFT** | r | 0.203 | 0.264 | -0.154 | -0.204 | 0.041 | 0.134 |
|  | p_adj | 0.542 | 0.542 | 0.635 | 0.542 | 0.9 | 0.651 |
| **FAB** | r | 0.047 | -0.065 | -0.198 | 0 | -0.227 | -0.227 |
|  | p_adj | 0.889 | 0.823 | 0.542 | 1 | 0.635 | 0.542 |
| **ROCF copy** | r | 0.027 | -0.107 | -0.172 | -0.041 | -0.088 | -0.246 |
|  | p_adj | 0.915 | 0.732 | 0.635 | 0.891 | 0.823 | 0.542 |

**Supplementary Table 4**. Correlations between cognitive and kinematic variables. P-values adjusted for false discovery rate (FDR). Significant values are in bold. Please note that a positive correlation was also observed between finger tapping velocity and interference memory at 10 and 30 seconds, revealing correlation coefficients of 0.3 (p=0.008) and 0.2 (p=0.03), respectively, although these were not significant after false discovery rate (FDR) correction. CV: coefficient of variation. MoCA: Montreal Cognitive Assessment, BSRT: Babcock Story Recall Test, RAVLT: Rey-Auditory Verbal Learning Test, ROCF: Rey–Osterrieth complex figure, TMT-A: Trail Making Test-A, TMT-B: Trail Making Test-B, TMT-BA: Trail Making Test B – A, SCWT: Stroop Color and Word Test, VFT: Verbal Fluency Test, FAB: Frontal Assessment battery.

**SUPPLEMENTARY FIGURE**

**

**

**Supplementary Figure 1. Correlation analysis in patients with essential tremor (ET).** BSRT: Babcock Story Recall Test. Finger tapping movement velocity is expressed in degrees/sec.
